# *De novo* ssRNA Aptamers against the SARS-CoV-2 Main Protease: In Silico Design and Molecular Dynamics Simulation

**DOI:** 10.3390/ijms22136874

**Published:** 2021-06-26

**Authors:** Francesco Morena, Chiara Argentati, Ilaria Tortorella, Carla Emiliani, Sabata Martino

**Affiliations:** 1 Department of Chemistry, Biology and Biotechnology, University of Perugia, Via del Giochetto, 06123 Perugia, Italy; chiara.argentati@unipg.it (C.A.); tortorellailaria@gmail.com (I.T.); carla.emiliani@unipg.it (C.E.); 2 Centro di Eccellenza CEMIN (Materiali Innovativi Nanostrutturali per Applicazioni Chimica Fisiche e Biomediche), University of Perugia, 06123 Perugia, Italy

**Keywords:** COVID-19, single strand RNA aptamer, prediction of 3D RNA aptamer structure, aptamer-protein interaction, aptamers virtual screening, aptamer-protein free energy calculation

## Abstract

Herein, we have generated ssRNA aptamers to inhibit SARS-CoV-2 M^pro^, a protease necessary for the SARS-CoV-2 coronavirus replication. Because there is no aptamer 3D structure currently available in the databanks for this protein, first, we modeled an ssRNA aptamer using an entropic fragment-based strategy. We refined the initial sequence and 3D structure by using two sequential approaches, consisting of an elitist genetic algorithm and an RNA inverse process. We identified three specific aptamers against SARS-CoV-2 M^pro^, called MApta^pro^, MApta^pro^-IR1, and MApta^pro^-IR2, with similar 3D conformations and that fall in the dimerization region of the SARS-CoV-2 M^pro^ necessary for the enzymatic activity. Through the molecular dynamic simulation and binding free energy calculation, the interaction between the MApta^pro^-IR1 aptamer and the SARS-CoV-2 M^pro^ enzyme resulted in the strongest and the highest stable complex; therefore, the ssRNA MApta^pro^-IR1 aptamer was selected as the best potential candidate for the inhibition of SARS-CoV-2 M^pro^ and a perspective therapeutic drug for the COVID-19 disease.

## 1. Introduction

Currently, there is no available therapy for the Coronavirus Disease 2019 (COVID-19), a pandemic caused by a novel Severe Acute Respiratory Syndrome Coronavirus 2 (SARS-CoV-2) that began in December 2019 and accounted for more than 3.4 million deaths of infected patients in the world (as of May 2021) [1]. In a short time, considerable efforts have been made worldwide to develop strategies to combat the disease progression through a combination of drugs already used for other virus pandemics, or with new molecules being discovered [2,3,4]. In the meantime, efforts have been made to produce several vaccines to prevent disease infection [5,6,7,8]. However, this hope has been tempered mostly by the absence of an effective antiviral drug as well as by logistical and vaccine manufacturing troubles. Additionally, the current discovery of new variants of SARS-CoV-2 [9,10] raises more questions about the virus epidemiology in the scientific community.

The SARS-CoV-2 is a single-stranded RNA virus that shares ~80% and ~50% sequence identity with the other *coronaviridae* SARS-CoV and MERS-CoV [11], respectively. The SARS-CoV-2 genome consists of 14 open reading frames (ORF) that encode for the 4 structural proteins (spike [S], envelope [E], membrane [M], and nucleocapsid [N]), 9 accessory proteins, and the 16 non-structural proteins (NSPs) [12]. The spike protein binds specifically to the angiotensin-converting enzyme 2 (ACE2) receptor of the cells, and, by this, mediates the entry of the SARS-CoV-2 virus into the cells. It was suggested that ACE2 binds the S1 subunit of the spike protein at the receptor-binding domain and that this interaction causes the S2 subunit conformational change, which facilitates the internalization process of SARS-CoV-2, also with the help of cathepsin L and the transmembrane protease serine 2 [13,14,15,16].

Within the cells, the viral genome is first translated into the NSP proteins. The latter are encoded by the ORF1a (NSPs 1 to 11) and ORF1ab (NSPs 1 to 16) genes, translated into polyproteins and processed into individual NSPs through viral proteases. Among NSPs, the SARS-CoV-2 main protease, also known as 3-chymotrypsin-like cysteine protease (CCP or 3CLpro), and now M^pro^, is encoded by the NSP5 gene and is the main viral protease responsible for the processing of polyproteins [17]. M^pro^ is involved in the generation of 12 NSPs, from NSP4 to Nsp16, and also includes the RNA-dependent RNA polymerase (Nsp12) and the helicase (Nsp13); thereby, it has a key role in the viral replication process and the death of the host cells. M^pro^ has three domains: domains I (residues 8–101), II (residues 102–184), and III (residues 201–303), with the Cys-His catalytic site, and the substrate-binding site located in a gap between the domain I and domain II [18]. The X-ray crystallographic structure of M^pro^ showed that this protease can exist in both monomeric and dimeric composition but only the dimeric state is functional [18]. Notably, M^pro^ stands out as a highly conserved gene (96% sequence identity among the SARS-CoV and SARS-CoV-2); therefore, it is a good candidate for developing effective molecules against SARS-CoV-2 and the other coronavirus variants [19,20,21,22,23]) that can be frequent in the spike protein [10]. For instance, of the widespread infective variants, S-D614G seems to cause a conformational change of the spike protein with a consequent binding outside of the receptor-binding domain and increase of the stability of the binding with receptor ACE2.

Here, we aim to develop a strategy for the inhibition of the SARS-CoV-2 M^pro^ activity, and the consequent blocking of the viral replication. Up to now, the availability of the crystallized structure of the SARS-CoV-2 M^pro^ in the Protein Data Bank [24] has increased the development of inhibitors, such as 11a and 11b compounds, the more recent synthesized inhibitor Ac-Abu-DTyr-Leu-Gln-VS [25], and some natural compounds [26,27,28,29]. However, no inhibitors targeting the substrate-binding pocket have reached clinical trials to date.

In this work, we are proposing aptamer technology as a valuable approach to predicting specific inhibitors of the SARS-CoV-2 M^pro^ enzyme.

Aptamers are short single-stranded DNA or RNA oligonucleotides (usually from 20 to 80 nucleotides) that tether proteins with high affinity due to their unique three-dimensional conformation [30]. These molecules are specifically designed to recognize and bind to the molecular targets and induce a peculiar biochemical effect (e.g., inhibition, activation, denaturation). From these characteristics, aptamers recapitulate the monoclonal antibody specificity, but also offer the advantages of smaller physical size, high stability, flexible and chemical modifiable structure, lack of immunogenicity, fast and low-cost production, and increasingly unlimited applications [31,32].

Currently, aptamers can be synthesized through the systematic evolution of ligands by exponential enrichment (SELEX) technique, an in vitro process that aims at the isolation and amplification of the nucleic acid candidates with the highest specificity and affinity from a nucleic acid library comprising a high number of different sequence strands with the same length. Additionally, aptamers’ oligonucleotide sequences can be easily modified and engineered into so-called aptamer–drug conjugates [33]. Moreover, due to the high specificity, they may bind to the cell membrane receptors, thus serving as ideal targeting drug delivery systems. Currently, several aptamers have been selected as candidates for the diagnosis and therapy for several diseases (e.g., cancer, acquired immune deficiency syndrome, AIDS, hepatitis, tuberculosis, Zika, Ebola) [34].

The design of the library and selection are the main critical steps for the generation of functional aptamers. In this regard, the computational approach represents a powerful tool for the prediction and validation of aptamers in silico [35].

In this study, we used the computational approach to develop single-stranded (ss)RNA aptamers against the SARS-CoV-2 M^pro^ protein which could thereby block the SARS-CoV-2 viral replication. We have designed *de novo* ssRNA aptamers against SARS-CoV-2 M^pro^ in silico, and have optimized the primary sequences and the conformation stability to refining the binding properties, and to select the aptamer against SARS-CoV-2 M^pro^ with the highest inhibition potential.

## 2. Results

### 2.1. Design of Aptamers against M^pro^

Currently, there are no available aptamers against M^pro^ protein in databanks or the literature; therefore, we have designed and generated a *de novo* single strand (ss)RNA aptamer against the M^pro^ protein; then, we have used this aptamer as a model to generate aptamers with the highest specificity toward the M^pro^ protein.

**Figure 1:**
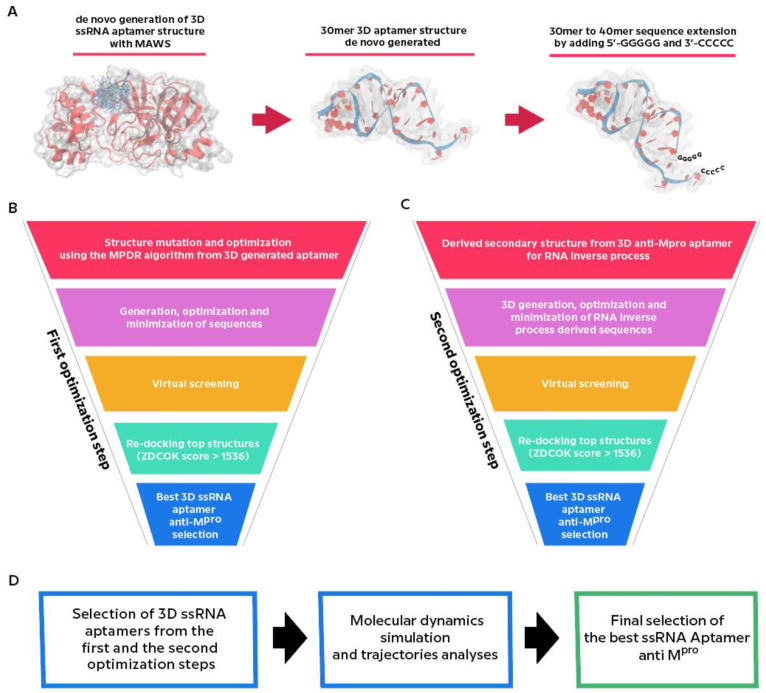
Schematic of the workflow for the anti-M^pro^ ssRNA aptamers. (**A**) initial 30mer 3D ssRNA aptamer generation. (**B**) First optimization step. (**C**) Second optimization step. (**D**) Schematic of Molecular Dynamics simulation and trajectories analyses and best aptamer selection. See details in the text.

Figure 1 schematizes the workflow for the production of the against-M^pro^ ssRNA aptamer.

First, we generated *de novo* an initial 30mer 3D ssRNA aptamer structure against M^pro^ by Making Aptamers Without SELEX algorithm (MAWS) [36] (Figure 1A). The obtained structure was extended to 40mer by adding 5′-GGGGG and 3′-CCCCC sequences (Figure 1A) and subjected to the first optimization step (Figure 1B), consisting of sequence mutation and optimization by using the Mutate Predict Dock Repeat (MPDR) algorithm [37], virtual screening and re-docking analyses, and selection of the best ssRNA aptamer structure (Figure 1B). The latter was subjected to the second optimization step (Figure 1C), consisting of the RNA inverse process optimization, virtual screening and re-docking analysis, and selection of the best ssRNA aptamers based on the concordance of scores between software.

The best aptamer structures selected from the first and second optimization steps were subjected to molecular dynamics (MD) simulations in complex with M^pro^ (Figure 1D). The MD simulation trajectories were used as inputs for the calculation of binding free energies of the aptamers and the investigation of their binding mechanism for the final selection of the best ssRNA aptamer against M^pro^ (Figure 1D).

### 2.2. Generation of De novo ssRNA Aptamer and First Optimization Step

**Figure 2:**
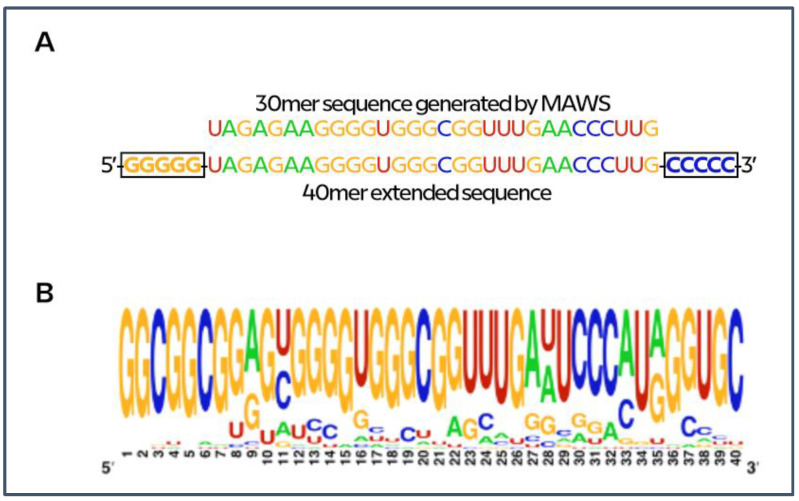
(**A**) Originated 30mer MAWS sequence extended to 40mer before elitist genetic algorithm mutation process (MPDR). (**B**) Weblogo image that shows residue frequencies of generated (40mer) sequences from 30mer starting sequence (https://weblogo.berkeley.edu/logo.cgi, February 2021).

Many bioinformatics approaches have been carried out to discover novel aptamers [38,39], as well as to probe and assess the aptamer–protein interactions [40,41,42]. We used the iGEM MAWS algorithm [36] with some personal modifications (see method Section 4.1) to produce a 30mer ssRNA aptamer (Figure 2A) specific for the M^pro^ crystallography structure (PDB #6LU7) [18]. This RNA sequence was used to evolve the 30mer aptamer to a final length of 40mer by adding 5’-GGGGG and 3’-CCCCC sequences to the aptamer terminal ends (Figure 2A). The length of 40mer allowed the generation of an aptamer with a suitable size for covering the designed target region of M^pro^.

Next, we performed the first optimization step by using the MPDR algorithm. From the 40mer RNA aptamer (Figure 2A), 200 mutated and optimized sequences were generated, which were then used to create a logo representation (Figure 2B). The logo representation showed that the majority of generated sequences were stable and not skewed in favor of some of the other ssRNA (Figure 2B). All 200 sequences (ssRNAs) were converted to the co-respective 3D structures through SimRNA 3D and optimized with QRNAS algorithms through MPDR, and the latter were used as ligands to predict their binding specificity toward the M^pro^ enzyme by molecular docking analysis.

#### Molecular Docking Simulation of *De novo* ssRNA Optimized Aptamer against M^pro^

The identification of the best *de novo* 3D ssRNAs aptamer was performed by three different docking platforms: ZDOCK, HDOCK, and QVINA-W. Only aptamers showing favorable and equivalent binding modes in all three tools were considered as actives. ZDOCK was used as the first step to dimensionally reduce the 200 ssRNA aptamer structures to the best 10 because it is faster in the screening process due to its algorithm, and it gives the possibility of parallel execution. Thus, we performed a docking analysis using a set of 13 random X-ray-resolved ssRNA aptamer structures, stripped from the protein complexes in the PDB database (PDB.org, February 2021; Table S1), and set up the threshold value of ZDOCK score, docking these structures against M^pro^.

From the first best pose of each docked 13 X-ray structure (Table S1), we calculated the ZDOCK mean, which corresponded to 1370 ± 83, and by this, we have set the threshold ZDOCK score to 1536 (1370 + 2SD). Two hundred docking experiments using ZDOCK algorithms were carried out to predict the most probable aptamer in the collected sets of structures, and the top 10 aptamer-M^pro^ complexes were selected for a further re-docking process step with HDOCK and QVINA-W (Table 1).

**Figure 3:**
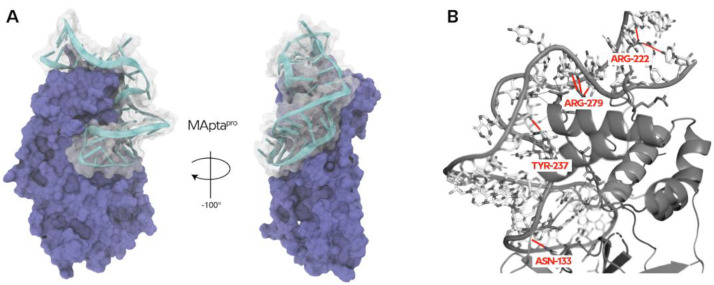
(**A**) VMD generated image of 3D-40mer aptamer MApta^pro^ in complex with the protein M^pro^. (**B**) PyMol generated image of hydrogen bonds of the best docked pose of the selected aptamer MApta^pro^ (for detailed interaction see Figure S1 in Supplementary file).

The ZDOCK top 10 aptamers were re-docked integrating HDOCK and QVINA-W algorithms and, based on (i) the concordance of the three docking algorithms scores, and (ii) the same pose predicted by all algorithms (Table 1), the sequence 5′-GGCGGCGGAGCGGGGUGGGCGGUUUGAAUCCCAUAGGUGC-3′ was identified as the best one that gives rise to the most specific 3D ssRNA structure against M^pro^. We call this aptamer MApta^pro^ (Figure 3A).

The identified pose had the lowest docking energy with a score of 1738.34, −340, and −17.4 (ZDOCK, HDOCK, and QVINA-W, respectively) (Table 1), almost due to the four hydrogen bonds with ASN-133, ARG-222, TYR-237, and ARG-279 (Figure 3B) (distance below 3Å between donor and acceptor atoms; for detailed interaction see Figure S1 in the Supplementary File), and two hydrophobic interactions with ASN-221 and ARG-222 (Figure S1).

### 2.3. Generation of the 3D ssRNA Aptamer Structures Starting from MApta^pro^ Sequence: Second Optimization Step

Next, we used the above-described aptamer MApta^pro^ to generate aptamers with the highest binding specificity toward the M^pro^ protein. We have chosen the elitism-based algorithm in MPDR in the interest of time, even if it can be trapped in local optima.

**Figure 4:**
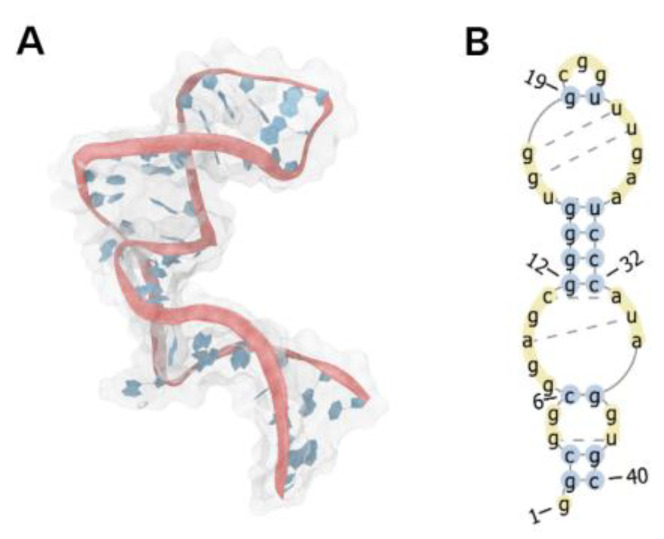
(**A**) Generated image of MApta^pro^ with VMD software. (**B**) Derived secondary structure topology resolved and encoded by Elimination Max-Conflicts algorithm. Image generated by PseudoViewer-based procedure (RNApdbee).

To mitigate this event, the secondary structure was derived from the best pose of the MApta^pro^ aptamer (Figure 3 and Figure 4A,B) and subjected to an inverse RNA folding process (Figure 1C) to predict the sequences that fold into a target minimum free energy secondary structure, taking into account several constraints.

#### 2.3.1. Inverse RNA Processing

Inverse RNA folding is traditionally designed to optimize RNAs with favorable properties. Using an RNAinverse web server [43,44], we obtained 207 sequences (Supplementary file “Output of RNAinverse WebServer”) that were post-processed with the pipeline used for the generation of the above described ssRNA MApta^pro^ aptamer (Figure 1). In this process, we used the ViennaRNA algorithm for secondary structure prediction in favor of the energy minimization, the SimRNA algorithm for the prediction of the 3D structure from the primary sequence and the secondary structure, and the QRNA algorithm for finally refining and minimize the 3D structure of aptamer for subsequent docking.

#### 2.3.2. Molecular Docking Simulation of 3D ssRNA Aptamer Structures

We performed molecular docking according to the previous workflow (Figure 1C). Hence, after dimensional reduction with ZDOCK docking of the sequences generated from the first aptamer MApta^pro^, the best structures were re-docked with HDOCK, and QVINA-W, and chosen based on (i) the concordance of the three docking algorithms scores, and (ii) the same pose predicted by all algorithms.

We identified only two structures with a score over the ZDOCK threshold value of 1536 as probable aptamers for the M^pro^ protein. Both structures have shown a better ZDOCK score compared to MApta^pro^ aptamer but a lower score in HDOCK and QVINA-W (Table 2).

**Figure 5:**
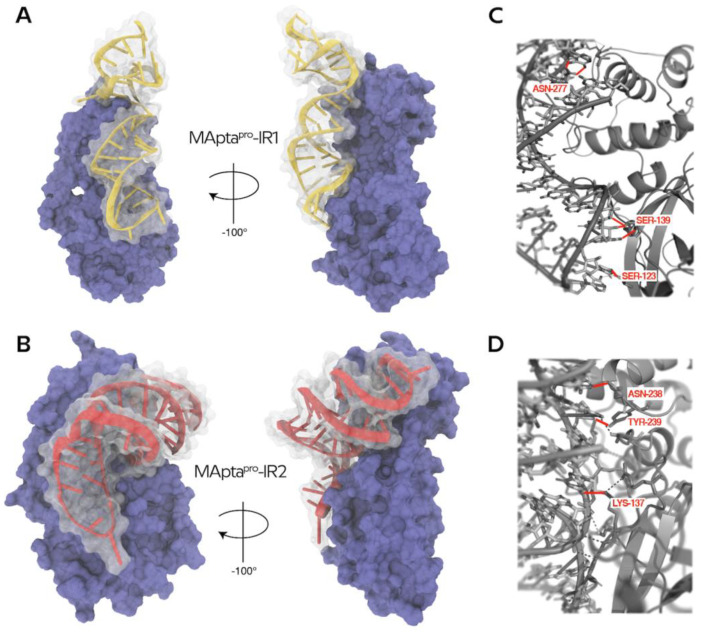
(**A**,**B**) VMD generated image of 3D-40mer aptamers MApta^pro^-IR1 and MApta^pro^-IR2, respectively, in complex with the protein M^pro^. (**C**,**D**) PyMol generated image of hydrogen bonds of the best docked pose of the selected aptamers MApta^pro^-IR1 and MApta^pro^-IR2, respectively (for detailed interaction see Figures S2 and S3 in the Supplementary File).

The aptamers were called MApta^pro^-IR1 (GGCGGCGAAACGGAGCUCCCAGGGAAUGGUCCAAAGGCGC) and MApta^pro^-IR2 (GGCGGUGGAAAGGGGAAUGCGGCAUGAUGCCCGUAGGUGC), and related structures are reported in Figure 5A,B.

The ssRNA aptamers MApta^pro^-IR1 and MApta^pro^-IR2 have the lowest docking energy of −15.69 kcal/mol and −13.61 kcal/mol, respectively (Table 2) and formed three hydrogen bonds that resulted importantly in tethering with the M^pro^. Specifically, the MApta^pro^-IR1 aptamer arranged hydrogen bonds with SER-123, SER-139, and ASN-277 residues of M^pro^ (Figure 5C and Figure S2), and the MApta^pro^-IR2 aptamer engaged hydrogen bonds with LYS-137, ASN-238, and TYR-239 residues of M^pro^ (Figure 5D and Figure S3). No hydrophobic interactions were found (Figures S2 and S3).

**Figure 6:**
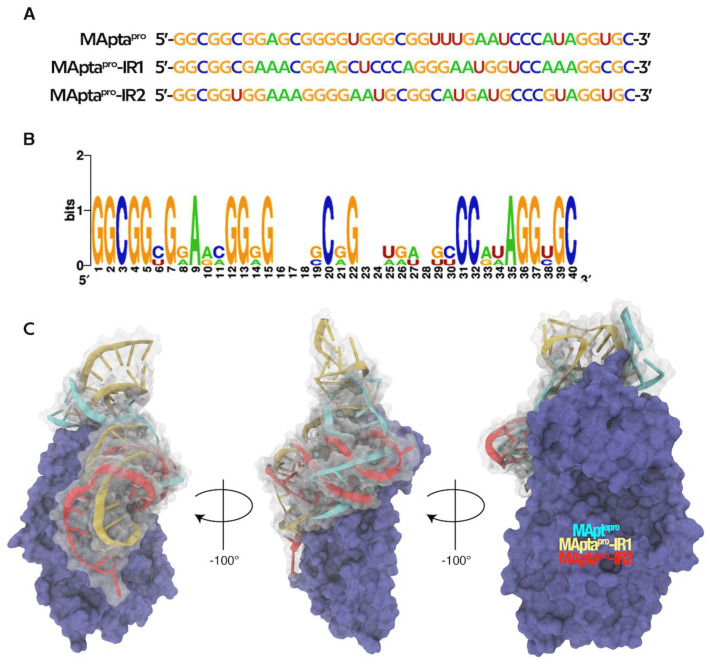
(**A**) Sequences of the best selected ssRNA aptamers. (**B**) Weblogo of the three sequences of the best selected aptamers. (**C**) VMD generated image showing the overlap of MApta^pro^ (cyan), MApta^pro^-RI1 (yellow) and MApta^pro^-RI2 (red) aptamers in complex with M^pro^ (purple).

Thus, from the original MApta^pro^, the InverseRNA approach generated two ssRNA aptamers with similar 3D conformations, comparable docking scores and that were favorable to forming the complex with the protein. Notably, MApta^pro^-IR1 and MApta^pro^-IR2, as well as MApta^pro^, fall in the same region of M^pro^ (Figure 6).

### 2.4. Molecular Dynamics Simulations and Trajectory Analyses of MApta^pro^, MApta^pro^-RI1, and MApta^pro^-RI2 Aptamers

We used the AMBER software to ensure the efficacy interaction of MApta^pro^, MApta^pro^-IR1, and MApta^pro^-IR2 aptamers with the M^pro^ protein. The simulation time of the dynamic stability process was over 100 ns. We applied canonical analytical methods to analyze the structural stability of biomacromolecules in molecular dynamics (MD) simulation: (i) root-mean-square deviation from the initial simulation structure; (ii) the radius of gyration identifying the structure stability and compactness of modeled conformation (iii) the number of hydrogen bonds, and (iv) the binding free energy analysis.

#### 2.4.1. The Root-Mean-Square Deviation (RMSD)

**Figure 7:**
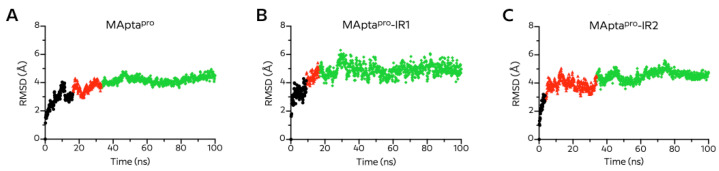
Time dependence of root-mean-square deviation (RMSD) for (**A**) MApta^pro^, (**B**) MApta^pro^-IR1, (**C**) MApta^pro^-IR2 in complex with M^pro^ protein during 100 ns molecular dynamics simulation and respect to the first frame. Black, red and green points represent clustering structures in PC space of conformational differences derived from principal components analysis of trajectories (Figure S4A–C).

The RMSD plot of the three aptamers, and principal component analysis (PCA), revealed some differences among the aptamer structures during the 100 ns simulation time (Figure 7A and Figure S4). MApta^pro^ had the highest RMSD of 4.37Å with an average of 3.37 ± 0.64Å and showed some fluctuations. The aptamer–protein interaction attempts to reach an equilibrium state with an oscillation of 3Å after 12 ns. This new state was lost after 5 ns of simulation, as the RMSD increased steadily until 30 ns when reaching a new equilibrium state (Figure 7A). The equilibrium state was observed after 20 ns with a fluctuation of 4Å and 3Å for both MApta^pro^-IR1 and MApta^pro^-IR2 complexes, respectively. The highest RMSD values for MApta^pro^-RI1 was 5.40Å with an average of 4.25 ± 0.66Å, while the MApta^pro^-RI2 RMSD was 4.04Å with an average of 3.14 ± 0.43Å. In both cases the equilibrium state continued over the simulation time (Figure 7B,C).

#### 2.4.2. The Radius of Gyration (Rg)

**Figure 8:**
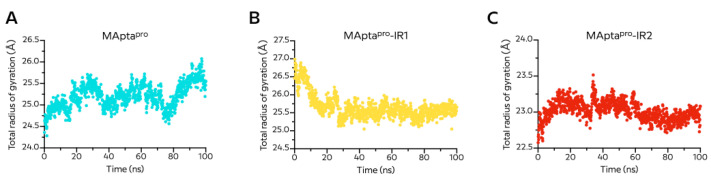
Time evolution of the radius of gyration (Rg) for (**A**) MApta^pro^, (**B**) MApta^pro^-IR1, and (**C**) MApta^pro^-IR2 in complex with M^pro^ protein during 100 ns molecular dynamics simulation.

The MApta^pro^/M^pro^ Rg values were more fluctuating, with an average of 25.11 ± 0.26 Å, and confirmed the reduced stabilization of the process during the simulation time of 100 ns and bared equilibrium (Figure 8A), as shown by RMSD analysis.

The Rg trajectory plot of the MApta^pro^-IR1/M^pro^ complex was stable after 20 ns, and the oscillation values were negligible (Figure 8B). After that, the complex was at equilibrium and confirmed the steady interaction between the aptamer and the protein. This value remained virtually unchanged during the simulation time with an Rg average of 25.94 ± 0.35 Å for the MApta^pro^-RI1/M^pro^ complex (Figure 8B).

Finally, the MApta^pro^-IR2/M^pro^ complex had an Rg average of 23.04 ± 0.13 Å and was stable after 20 ns with a slight oscillation around 35 ns (Figure 8C).

#### 2.4.3. Number of Hydrogen-Bonds

**Figure 9:**
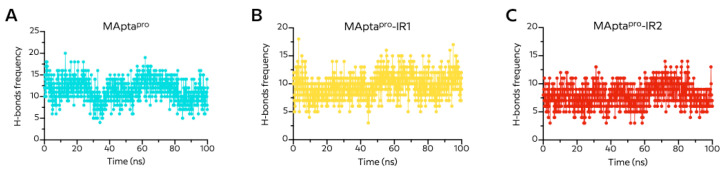
Total number of hydrogen bonds of (**A**) MApta^pro^, (**B**) MApta^pro^-IR1, (**C**) MApta^pro^-IR2 in complex with M^pro^ protein during 100 ns molecular dynamics simulation.

The number of intramolecular H-bonds formed is a measure of the aptamer–protein interaction stability. The total number of H-bonds from MApta^pro^, MApta^pro^-IR1, and MApta^pro^-IR2 in complex with the main protease M^pro^ is shown in Figure 9. During the simulation time, the number of H-bonds in the MApta^pro^/M^pro^ complex decreased, while the number of stable H-bonds in the MApta^pro^-IR1/M^pro^ and MApta^pro^-IR2/M^pro^ complexes remained nearly unchanged. The average number of H-bonds was 11.46 ± 2.59 for MApta^pro^, with the lowest number being four H-bonds, while the average number of H-bonds was 9.67 ± 2.13 for MApta^pro^-IR1, with the lowest number being three H-bonds, and was 7.96 ± 1.98 with the lowest number being three H-bonds for MApta^pro^-IR2 (Figure 9, Table 3).

### 2.5. Binding Free Energy Analysis of MApta^pro^, MApta^pro^-RI1, and MApta^pro^-RI2 Aptamers

**Figure 10:**
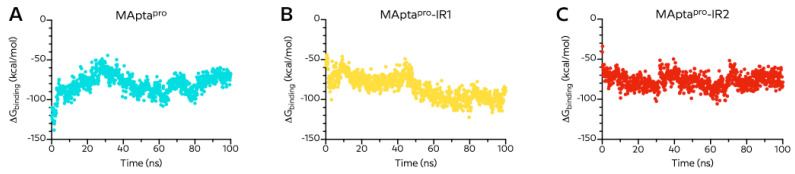
MM/PBSA binding energies vs. time for MApta^pro^ (**A**), MApta^pro^-IR1 (**B**), and MApta^pro^-IR2 (**C**) towards SARS-CoV-2 main protease (M^pro^).

Next, we assessed the relative binding free energies (ΔG_binding_) between the aptamers and M^pro^ enzyme by using the Molecular Mechanics/Poisson–Boltzmann Surface Area (MM/PBSA) method, often applied for the protein–ligand binding free energy calculation (Figure 10). We also assessed the entropy contribution with the Nmode AMBER module (Table 4).

The median value of ΔG_binding_ was −80.30 ± 12.73 kcal/mol for MApta^pro^, −85.03 ± 14.49 kcal/mol for MApta^pro^-IR1/M^pro^, and −77.18 ± 8.99 kcal/mol for MApta^pro^-IR2/M^pro^ (Figure 10). The trajectories of the MApta^pro^-M^pro^ interaction indicated an increase in the ΔG_binding_ during the simulation time, and higher stable ΔG_binding_ energy for the MApta^pro^-IR1/M^pro^ complex compared to MApta^pro^-RI2/M^pro^ and MApta^pro^/M^pro^.

Interestingly, while the ΔG_binding_ of the MApta^pro^/M^pro^ complex slightly increases in the last 20 ns of the trajectory and MApta^pro^-IR2/M^pro^ almost remain constant, in the MApta^pro^-RI1/M^pro^ complex, the ΔG_binding_ decreases from −57.74 kcal/mol to −92.25 kcal/mol (Figure 10B), indicating that the interaction between the MApta^pro^-IR1 aptamer and the M^pro^ enzyme is the strongest and the most stable.

We also calculated the MM/PBSA entropic contributions, TΔS, at 300 K, and combined these results with the MM/PBSA binding energies. Due to the expense of computing the entropic contributions with the Nmode AMBER module, we calculated the above-mentioned energies on five snapshots extracted from the representative structure calculated by clustering the trajectory. The representative structure corresponds to a representative snapshot from the most populated cluster. From this, we established the relative order of favorable binding energies: MApta^pro^-IR1 > MApta^pro^-IR2 > MApta^pro^ (Table 4).

Collectively, these data suggest that the most energetically favorable complex was the MApta^pro^-IR1 aptamer/M^pro^ protein (Video S1).

## 3. Discussion

In the present work, we have generated *de novo* ssRNA aptamers against the SARS-CoV-2 M^pro^ protein and demonstrated the formation of a stable aptamer–protein complex in a computational approach. We selected the SARS-CoV-2 M^pro^ as the target of our study based on its key role in the replication of the SARS-CoV-2 virus within infected cells [45], as well as by the databank information showing the highly-conserved SARS-CoV-2 M^pro^ sequence among other *coronaviridae*, such as SARS-CoV and MERS-CoV, and the absence of viral mutations, to our knowledge, mostly documented in the spike gene [46].

Due to these characteristics, SARS-CoV-2 M^pro^ is currently an attractive antiviral candidate, and many molecular drugs with potential antiviral activity are under therapeutic evaluation. These include phytochemicals compounds (e.g., Bonducellpin D. [47]; glycyrrhizin, tryptanthrine, indirubin, β-sitosterol, hesperetin, β-caryophyllene [48]), synthesized lead compounds 11a and 11b [24] and Ac-Abu-DTyr-Leu-Gln-VS [25], and molecular drugs (e.g., boceprevir, calpain inhibitor II, calpain inhibitor XII, GC-376 [49]; ceftaroline fosamil and telaprevir [50]; GRL-1720 and 5 h [51]). Most of these inhibitors have been identified by using bioinformatics virtual screening approaches, since many learning platforms have been developed, published, and hourly updated [52,53] to provide a suitable tool for improving the development of an effective therapeutic approach for the SARS-CoV-2 pandemic. Currently, there is no cure for the SARS-CoV-2 disease.

We chose ssRNA aptamers as antiviral drugs based on their chemical composition and 3D conformation that holds them as natural compounds for clinical purposes [39,54,55,56,57,58,59,60,61]. In particular, aptamers, similarly to monoclonal antibodies, interfere specifically with the target and block its function; furthermore, they can be easily transferred to cells using a conventional drug delivery system [62] and can be produced at low costs through a synthetic process. The highest critical step, consisting of the identification of the aptamer with the highest affinity for the target, is generally solved with the SELEX procedure or through the computational approaches [35].

In our study, we used the computational strategy for predicting the aptamer against the SARS-CoV-2 M^pro^ specific sequence, and for identifying the aptamer(s) with the highest affinity by dynamic molecular simulation. Up to now, there are no available aptamers active against SARS-CoV-2 M^pro^ in the databanks or published reports; therefore, as the first step of our work, we have designed *de novo* an aptamer specific for SARS-CoV-2 M^pro^. To the best of our knowledge, there are only two Korean patents that describe the generation of RNA aptamers for the inhibition of the SARS helicase enzyme (#KR2009128837) and those of nucleocapsid of SARS-CoV (#KR2012139512), whereas DNA aptamers targeting the receptor-binding domain (RBD) of the SARS-CoV-2 spike protein have been discovered by using the competition-based selection strategy and a machine learning screening algorithm [63].

The first ssRNA aptamer (MApta^pro^) was designed considering as reference the crystallography of SARS-CoV-2 M^pro^ [18]. From MApta^pro^, by the RNAinverse process, 207 ssRNA aptamers were generated, which, after molecular docking, allowed the selection of the aptamers MApta^pro^-IR1 and MApta^pro^-IR2 as the structures with the best docking energy. Notably, MApta^pro^, MApta^pro^-IR1, and MApta^pro^-IR2 have a similar 3D conformation and almost fall in the same structural region of SARS-CoV-2 M^pro^, that is, the dimerization region of the protein necessary for the enzymatic activity [45]. Furthermore, dynamic molecular simulation and the free binding energy calculation have shown that the three aptamers formed a stable complex with the SARS-CoV-2 M^pro^ and highlighted that the complex MApta^pro^-IR1/SARS-CoV-2 M^pro^ was the most favorable. We speculated that this is the consequence of the capability of MApta^pro^-IR1 to form H-bonds with Arg4 and Ser139, whereas MApta^pro^ and MApta^pro^-IR2 form only H-bonds with Arg4. Both amino acids are part of the amino acid Arg4, Ser10, Gly11, Glu14, Asn28, Ser139, Phe140, Ser147, Glu290, Arg298 residues that are necessary for the dimerization process of SARS-CoV-2 M^pro^ monomers [45]. In particular, it is reported that Ser139 is one of the amino acids close to the binding site necessary for enzyme activity since its substitution impaired the dimerization process, thus inhibiting enzyme activity [45,64], whereas, Arg4 is necessary for the dimerization as its substitution causes a 5-fold decrease of this process [45,65].

The overall characteristics highlighted MApta^pro^-IR1 as the best potential candidate for the inhibition of SARS-CoV-2 M^pro^ and useful as a therapeutic drug for the COVID-19 disease.

To the best of our knowledge, our results are the pioneer for predicting ssRNA aptamers as molecular drugs against SARS-CoV-2 M^pro^ and, through this perspective, in combatting the SARS-CoV-2 pandemic.

## 4. Materials and Methods

### 4.1. De novo Generation of 3D ssRNA Aptamer Structure against M^pro^ of SARS-CoV-2

**Figure 11:**
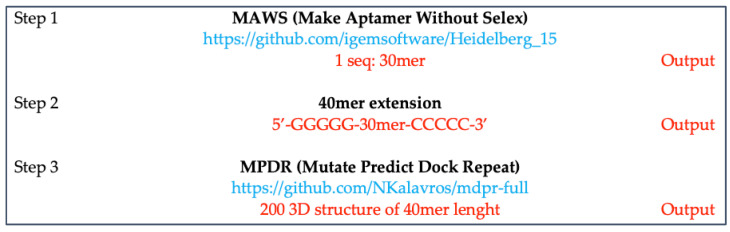
Schematic representation of the workflow.

MAWS is an entropic fragment-based algorithm that can create aptamers against target molecules without the need for an initial aptamer sequence or pool [36]. The MAWS algorithm was slightly modified in (1) forcefield name = “leaprc.protein.ff14SB” and (2) “residue.name” for RNA instead of DNA, to generate an RNA aptamer. The algorithm requires a target molecule to be loaded, and then a bounding box is created. The crystal structure of M^pro^ (PDB ID: 6LU7) is taken from Protein Data Bank [22,66]; it is cleaned and unwanted molecules are removed. Next, during the initial sampling, nucleotides are sampled from a uniform distribution, “docked” and the nucleotide with the lowest entropy is chosen as the starting point. Then, possible conformations of the next nucleotide in the sequence are sampled and the sequence with the lowest energy is chosen. This process is continued until the aptamer is designed (step 1, Figure 11). The MAWS software creates an aptamer of a maximum length of 30mer, so in step 2 the aptamer was extended to 40mer by adding 5′-GGGGG and 3′-CCCCC.

Starting from this 40mer aptamer sequence (Figure 11, step 2), the Mutate Predict Dock Repeat (MPDR) algorithm [37,67] was used to generate 200 mutated 3D structures of RNA aptamers. The original MPDR algorithm was only modified using ZDOCK, instead of an HADDOCK server, for its ability to run locally and rapidly on a personal computer.

All of the computations were performed on an Ubuntu18 desktop PC, with a [AMD Ryzen 9 5950x CPU (16 Cores) + 64GB RAM + 1 NVIDIA GeForce RTX 3090] configuration.

### 4.2. Generation of the 3D ssRNA Aptamer Structure from a Given Secondary Structure: RNA Inverse Process

**Figure 12:**
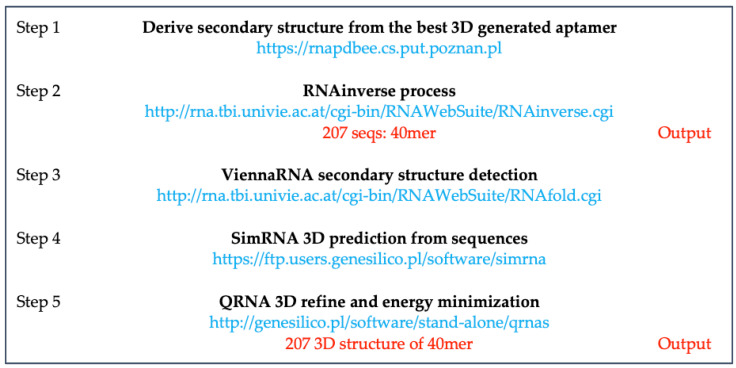
Schematic representation of the workflow.

Starting from the best *de novo* 40mer 3D aptamer, we also derive the secondary structure through RNAPDBEE [68,69] and submit then to the RNAinverse process by using the ViennaRNA web server [70,71] for finding a sequence that adopts a given secondary structure. The RNAinverse algorithm starts with a selected nucleotide sequence and keeps changing nucleotides until the RNA sequence adopts the desired fold. A total of 207 sequences were generated and the 3D structure was further modeled using ViennaRNA for secondary structure detection [44], SimRNA 3D [72] for structure prediction from the sequence, and QRNA 3D [73] for refinement and energy minimization (Figure 12).

### 4.3. Molecular Docking Simulation

All generated structures were subjected to virtual screening. For docking analysis, in this study, three different software docking algorithms—ZDOCK [74], HDOCK [75], and QVINA-W [76,77]—were used for predicting the structures of protein–RNA complexes.

Before docking, the crystal structure of M^pro^ (PDB ID: 6LU7) was taken from the Protein Data Bank [22,66], was cleaned and unwanted molecules were removed.

The latest version, ZDOCK 3.0.2 [78], was used in this study with the AMBER94 force field for improving accuracy in RNA–protein rigid-body docking [79]. Its docking procedure was performed with the default settings: variable grid size for fitting the size of a protein and RNA, a 1.2 Å grid step, with a 15° angle step for rotation of the ligand, and -N 5000 (number of output predictions).

The HDOCK server, for integrated protein–RNA and protein-protein docking [79] automatically predicts their interaction through a hybrid algorithm of template-based and template-free docking. The cleaned protein and RNA aptamer were loaded on the web server and docked with standard parameters.

To perform the docking with QVINA-W, the protein and RNA were prepared with AutoDockTools. For protein preparation, the standard script ‘prepare_receptor4.py’ was used, while for RNA preparation all active torsions were inactivated through ‘prepare_ligand4.py -Z’.

All of the docking simulations were performed on an Ubuntu18 desktop PC, with a [AMD Ryzen 9 5950x CPU (16 Cores) + 64GB RAM + 1 NVIDIA GeForce RTX 3090] configuration.

### 4.4. Molecular Dynamics Simulation

The MD simulation was carried out based on the complex obtained from docking with ZDOCK software. The complex using in the simulation was prepared with CHARMM-GUI [80].

Each simulation system was prepared in a periodic rectangular simulation cubic box, hydrated with a TIP3P [81] water model, and neutralized with a 0.15 M NaCl solution. The box dimensions were chosen to provide at least 30 Å buffers of solvent molecules around the solute.

All simulations were performed using the molecular dynamics software AMBER16 [82], the amber ff14SB force field for protein [83], and χ_OL3_ force fields for RNA [84]. This combination has shown satisfactory behavior with other protein–RNA complexes [85].

All systems were subjected to energy minimization and equilibration using a standard equilibration protocol [85]. Initially, each system was minimized by applying a maximum force of 5 kcal/mol/Å^2^ and 5000 steps steepest descent to remove clashes between atoms, followed by another 3 cycles of gradually relaxing minimization to 0.05 kcal/mol/Å^2^.

Before the simulation, each system was equilibrated through 3 cycles of 50 ps, gradually relaxing from 5 kcal/mol/Å^2^ to 0.1 kcal/mol/Å^2^ performed for each system at constant pressure (1 atm) and temperature (300 K). The temperature and pressure were kept constant during the simulation. Temperature coupling was done using the Berendsen thermostat with a temperature coupling constant of 0.5 ps, while the Berendsen barostat method was used for pressure coupling, with a reference pressure of 1 atm. The particle mesh Ewald approach [86,87] was used to calculate the long-range electrostatic, and a cut-off distance of 12Å was used for the van der Waals interactions (the non-bonded Lennard-Jones terms). The chemical bond lengths involving hydrogen atoms were constrained with the SHAKE algorithm. After equilibration, 100ns completely unrestrained MD simulations were performed.

All MD simulations were performed with a Linux16 image as a Google Compute Engine instance on Google Cloud Platform (8 vCPU, 30 GB RAM, GPU 1 × NVIDIA Tesla V100).

### 4.5. MD Simulation Analysis

To study the stability of the M^pro^-specific aptamer structures over the 100ns simulation time, various analytical methods were employed.

The trajectories of the MD simulations were examined with *cpptraj* implemented in AmberTools20 (e.g., RMSD, Radius of gyration, and H-bond frequency) [88]. Principal Component Analysis on trajectory was performed using the *bio3D* library in R v3.6.1 (R Core Team, Wien, Austria) [89,90,91].

The free binding energies of the complex between the aptamer and M^pro^ were analyzed during the 100 ns MD simulations with *MMPBSA.py* implemented in AmberTools20 [82,92]. The Molecular Mechanics/Poisson-Boltzmann Surface Area (MM/PBSA) [93,94] implicit solvent model is one of the most widely used methods to compute the binding free energy. In this work, the binding free energy, ΔG_binding_, is estimated from computational analysis of a single system simulation (only complex).

According to the MM/PBSA method, binding free energy (ΔG_binding_) is calculated by subtracting the free energies of the unbound receptor and ligand from the free energy of the bound complex:

Δ*G*binding,solvated = *G*complex,solvated − (*G*protein,solvated + *G*ligand,solvated) (1)

In addition, the binding free energy (Δ*G_binding_*) consists of two parts:

Δ*G*binding = Δ*G*gas + Δ*G*solv = (Δ*G*MM–TΔS) + Δ*G*solv (2)

Δ*G*MM = Δ*G*ele,int + Δ*G*VDW,int (3)

where Δ*G*ele,int and Δ*G*VDW,int represent the electrostatic and van der Walls energetic interactions, respectively.

The solvation free energy in Equation (2) can be expressed as:

Δ*G*solv = Δ*G*ele,solv + Δ*G*nonpolar,solv = Δ*G*PB + Δ*G*NP (4)

For each snapshot collected during the simulation, ligand–receptor interaction energies (Δ*G*MM) and the solvation energies (Δ*G*solv) were calculated with the MM/PBSA program implemented in AmberTools. The entropy contribution toward the binding free energy, TΔS, was computed by the AMBER NMODE (Nmode) module [95].

However, considering that it would be extremely time-consuming and computationally expensive to calculate the entropy contribution, we only extract 5 snapshots from the trajectory to calculate TΔS. The snapshots are extracted from the representative structure. A representative structure was calculated by clustering the trajectory using the amber module *cpptraj*. The representative structure corresponds to a representative snapshot from the most populated cluster.

### 4.6. Graphical Images

All the pictorial structure presentations were prepared using VMD-Visual Molecular Dynamics v1.9.4 (Theoretical and Computational Biophysics Group at the Beckman Institute for Advanced Science and Technology, University of Illinois, Urbana-Champaign) and PyMOL Molecular Graphics System v1.8.6.0 (Schrödinger, LLC, New York City, NY, USA). Molecular video of the 100ns trajectory of the selected best aptamer in complex with M^pro^ (Video S1) was prepared with UCSF Chimera v1.14 (developed by the Resource for Biocomputing, Visualization, and Informatics at the University of California, San Francisco, CA, USA). Graphical analysis was performed with GraphPad Prism 6 (GraphPad Software, La Jolla, CA, USA).

## 5. Conclusions

Herein, computational simulations probed three candidate ssRNA aptamer inhibitors of M^pro^. Two subsequential different approaches were used to optimize the initial sequence and its structure: (i) an elitist genetic algorithm that takes several potential solutions to a problem, creates offspring from them by adding variations (much like mutations), and then chooses the best solutions from the pool; and (ii) one followed by the RNA inverse process, which generates structure from a given sequence that folds into the target RNA structure.

We applied molecular docking, molecular dynamics, and binding free energy analysis to investigate the binding mechanism of several aptamers to SARA-CoV-2 M^pro^, and this is the first report to study the binding of an RNA molecule to the COVID-19 main protease. The screening of *de novo* designed aptamers retrieved 407 structures as candidate inhibitors of M^pro^.

The docking of candidate inhibitors filtered 12 structures with a docking ZDOCK score >1536 and established polar and non-polar interactions with the M^pro^ residues. Finally, molecular dynamics simulation and binding free energy analyses identified one candidate inhibitor of M^pro^ with the highest energy. During the 100ns simulation, the final candidate inhibitor established stable hydrogen-bond interactions with the M^pro^ Arg4 and Ser139 residues important for the dimerization and function of M^pro^ and obtained the lowest binding energies of −29.93 kcal/.

This study contributes to the understanding of how proteins binding with aptamer structures in an aqueous solution behave and may facilitate further infective drug design targeting the SARS-CoV-2 M^pro^. The workflow created allows for the development and testing of further attractive ligand–aptamer complexes.

## Figures and Tables

**Table 1:** Docking score of the best 10 *de novo* ssRNA aptamer against M^pro^ structures.

ID	ZDOCK Score	HDOCK Score	QVINA-W Score (kcal/mol)	Docking Hbond of QVINA-W Complex
MApta^pro^	1738.34	−340	−17.4	6
MApta1^pro^	1735.42	−264	−19.6	5
MApta2^pro^	1712.23	−258	−14.2	5
MApta3^pro^	1768.38	−246	−13.3	4
MApta4^pro^	1674.48	−268	−14.6	3
MApta5^pro^	1678.51	−265	−13.7	3
MApta6^pro^	1684.85	−266	−14.1	2
MApta7^pro^	1661.02	−261	−10.8	2
MApta8^pro^	1664.22	−258	−13.4	2
MApta9^pro^	1615.67	−254	−16.0	2

**Table 2:** Docking score of the best against M^pro^ aptamer structures generated with inverseRNA process.

ID	ZDOCK Score	Hdock Score	QVINA-W Score (kcal/mol)	Docking Hbond of QVINA-W Complex
MApta^pro^-IR1	1835.17	−290	−15.69	3
MApta^pro^-IR2	1756.89	−307	−13.61	3

**Table 3:** The table shows the results of the ‘hydrogen bonds’ interactions which show a percentage greater than one third of the trajectory (1/3 of 1000 frames, >330, i.e., > 33%).

ID_Complex	Acceptor	Donor	Occupancy (%)	Distance (Å)	Angle (°)
MApta^pro^	G_342@N3	ASN_274@ND2	52.6	2.88	158.71
	C_317@O2	ASN_277@N	49.8	2.85	153.89
	G_342@O2’	ASN_274@ND2	42.5	2.87	150.81
	C_346@OP1	ARG_222@NH2	41.8	2.78	158.71
	G_345@OP1	ARG_222@NH2	38.9	2.78	158.35
	G_328@OP2	ARG_4@NH1	38.5	2.78	159.16
	U_329@O2	ARG_4@NH2	36.6	2.84	151.91
	U_330@O4	ALA_285@N	34.6	2.86	162.63
	GLN_273@O	A_339@O2’	33.2	2.71	159.51
	C_317@O2’	GLY_278@N	33.0	2.90	155.15
MApta^pro^-RI1	U_323@OP2	ARG_4@NH2	89.9	2.78	161.16
	A_339@OP1	ARG_279@NH2	80.9	2.80	161.66
	C_322@OP1	ARG_4@NH2	73.1	2.81	158.14
	SER_139@O	G_318@N2	62.8	2.83	161.40
	A_339@OP2	ARG_279@NE	49.3	2.86	158.23
	ASN_274@O	G_329@N2	43.3	2.87	164.27
	G_328@O6	MET_276@N	42.6	2.87	161.74
	GLN_273@O	G_329@O2’	39.7	2.78	147.54
	U_323@OP1	ARG_4@NH1	38.6	2.84	159.93
	LEU_220@O	A_340@N6	34.0	2.89	159.49
MApta^pro^-RI2	G_335@OP1	TYR_118@OH	98.2	2.65	163.77
	A_333@OP1	SER_121@OG	70.2	2.68	163.72
	A_316@O2’	TYR_237@OH	70.1	2.82	160.94
	G_335@O4’	SER_123@OG	62.8	2.79	161.29
	G_318@OP1	MET_276@N	47.3	2.85	151.86
	C_338@OP1	ARG_4@NH2	36.3	2.79	155.40
	G_318@OP1	ASN_277@N	36.3	2.85	160.22
	THR_169@O	G_320@N2	34.3	2.84	153.97

**Table 4:** Summary of M^pro^-Aptamers binding affinities. The Nmode was used to compute the entropy contribution.

System	ΔG_gas_	ΔG_solv_	ΔG_PB Bind_	TΔS	ΔG_PB Bind_-TΔS
MApta^pro^/M^pro^	140.77 (2.15)	−215.31 (25.92)	−74.54 (0.56)	−72.62 (5.33)	−1.92 (5.89)
MApta^pro^-IR1/M^pro^	184.97 (3.11)	−281.22 (2.99)	−96.25 (0.54)	−66.32 (5.17)	−29.93 (5.71)
MApta^pro^-IR2/M^pro^	294.60 (2.12)	−369.64 (1.94)	−75.04 (0.52)	−69.65 (3.69)	−5.39 (4.21)

Data are reported as mean and SD of five snapshots extracted from the clustered representative structure. All units are reported in kcal/mole.

## Data Availability

Not applicable.

## Supplementary Materials

The following are available online at https://www.mdpi.com/article/10.3390/ijms22136874/s1.
