# Supplementary material for: De novo ssRNA Aptamers against the SARS-CoV-2 Main Protease: In Silico Design and Molecular Dynamics Simulation
Source: Int J Mol Sci. 2021 Jun 26;22(13):6874. doi: 10.3390/ijms22136874 (PMC8267631; doi:10.3390/ijms22136874)
Supplement: Supplementary file 1 [file ijms-22-06874-s001.zip › Morena_et_al_SupplementaryFile_MolecularInformatics.pdf]

**Supplementary File**

# *De novo* ssRNA aptamers against the SARS-CoV-2 Main Protease: in silico design and molecular dynamics simulation

**Francesco Morena [1*], Chiara Argentati [1], Ilaria Tortorella [1], Carla Emiliani [1,2] and Sabata Martino [1,2*]**

[1] Department of Chemistry, Biology and Biotechnology, University of Perugia, Via del Giochetto, 06123 Perugia, Italy; francesco.morena@unipg.it (F.M.); chiara.argentati@unipg.it (C.A.); tortorellailaria@gmail.com (I.T.); carla.emiliani@unipg.it (C.E); sabata.martino@unipg.it (S.M.).

[2] Centro di Eccellenza CEMIN (Materiali Innovativi Nanostrutturali per applicazioni Chimica Fisiche e Biomediche), University of Perugia, Perugia, Italy

* Correspondence: francesco.morena@unipg.it (F.M.), sabata.martino@unipg.it (S.M.); Tel.: +39-075-585-7442 (F.M, S.M.)

Supporting information file includes Supplementary Table1 and Figures S1-S4 with related captures.

**Table.S1** PDB ID of the 13 non-redundant Aptamer-protein crystal structures used for threshold estimation.

| **PDB IDs** |
|---|
| 1EXD 1Y26 2B57 2EES 2JWV 2L1V 2LUN 3GCA 3Q50 5KH8 6CF2 6E8S 6PQ7 |

**Figure.S1** PLIP interactions output of $MApta^{pro}$-$M^{pro}$ complex

Hydrophobic Interactions

| Index | Residue | AA | Distance | Ligand Atom | Protein Atom |
|---|---|---|---|---|---|
| 1 | 221A | ASN | 3.94 | 3175 | 1696 |
| 2 | 222A | ARG | 3.12 | 3175 | 1705 |

Hydrogen Bonds

| Index | Residue | AA | Distance H-A | Distance D-A | Donor Angle | Protein donor? | Side chain | Donor Atom | Acceptor Atom |
|---|---|---|---|---|---|---|---|---|---|
| 1 | 5A | LYS | 2.82 | 3.41 | 119.38 | ✓ | × | 33 [Nam] | 2851 [O3] |
| 2 | 5A | LYS | 3.20 | 3.71 | 114.19 | × | × | 2849 [O3] | 41 [O2] |
| 3 | 123A | SER | 2.68 | 3.44 | 135.48 | × | × | 2803 [O3] | 946 [O2] |
| 4 | 133A | ASN | 2.23 | 2.65 | 104.11 | ✓ | ✓ | 1021 [Nam] | 2694 [O2] |
| 5 | 138A | GLY | 2.68 | 3.47 | 137.73 | × | × | 2794 [Npl] | 1062 [O2] |
| 6 | 139A | SER | 2.37 | 3.30 | 161.97 | ✓ | ✓ | 1066 [O3] | 2785 [O3] |
| 7 | 197A | ASP | 1.79 | 2.65 | 144.08 | ✓ | × | 1499 [Nam] | 2692 [O3] |
| 8 | 197A | ASP | 2.67 | 3.22 | 115.89 | × | ✓ | 2695 [O3] | 1504 [O3] |
| 9 | 197A | ASP | 2.98 | 3.82 | 150.54 | ✓ | ✓ | 1504 [O3] | 2726 [O3] |
| 10 | 218A | TRP | 3.41 | 3.79 | 105.76 | × | × | 3159 [Nar] | 1674 [O2] |
| 11 | 218A | TRP | 2.86 | 3.37 | 113.07 | × | × | 3158 [Npl] | 1674 [O2] |
| 12 | 221A | ASN | 2.58 | 3.31 | 130.92 | ✓ | ✓ | 1699 [Nam] | 3164 [O3] |
| 13 | 222A | ARG | 1.51 | 2.46 | 160.70 | ✓ | ✓ | 1710 [Nox] | 2416 [N2] |
| 14 | 222A | ARG | 2.00 | 2.92 | 156.00 | ✓ | ✓ | 1709 [Npl] | 2394 [O2] |
| 15 | 222A | ARG | 3.44 | 3.88 | 109.45 | ✓ | × | 1702 [Nam] | 3177 [O3] |
| 16 | 222A | ARG | 3.18 | 3.87 | 128.19 | × | ✓ | 3195 [Npl] | 1707 [N2] |
| 17 | 237A | TYR | 2.43 | 2.99 | 117.85 | ✓ | ✓ | 1832 [O3] | 3061 [O3] |
| 18 | 270A | GLU | 3.00 | 3.65 | 127.76 | ✓ | ✓ | 2079 [O3] | 3141 [O3] |
| 19 | 272A | LEU | 2.29 | 3.20 | 153.43 | × | × | 2617 [Npl] | 2098 [O2] |
| 20 | 274A | ASN | 2.91 | 3.33 | 106.85 | ✓ | ✓ | 2113 [Nam] | 3141 [O3] |
| 21 | 274A | ASN | 3.15 | 3.52 | 104.08 | × | × | 2549 [Npl] | 2115 [O2] |
| 22 | 276A | MET | 3.00 | 3.98 | 173.75 | ✓ | × | 2120 [Nam] | 2607 [O3] |
| 23 | 277A | ASN | 3.30 | 4.09 | 138.55 | × | ✓ | 3136 [Nar] | 2132 [O2] |
| 24 | 277A | ASN | 2.32 | 3.02 | 126.69 | × | ✓ | 2506 [Npl] | 2132 [O2] |
| 25 | 277A | ASN | 3.30 | 3.81 | 113.58 | × | ✓ | 2507 [Nar] | 2132 [O2] |
| 26 | 277A | ASN | 2.49 | 3.19 | 127.73 | ✓ | ✓ | 2133 [Nam] | 2549 [Npl] |
| 27 | 278A | GLY | 2.95 | 3.71 | 136.11 | × | × | 2599 [O3] | 2136 [N2] |
| 28 | 279A | ARG | 2.89 | 3.80 | 153.51 | ✓ | ✓ | 2147 [Ng+] | 3136 [Nar] |
| 29 | 279A | ARG | 3.44 | 3.93 | 112.82 | × | ✓ | 3135 [Npl] | 2145 [Ng+] |
| 30 | 279A | ARG | 1.99 | 2.92 | 156.21 | ✓ | ✓ | 2148 [Ng+] | 2496 [O3] |
| 31 | 279A | ARG | 3.12 | 4.06 | 162.13 | × | ✓ | 2488 [O3] | 2145 [Ng+] |
| 32 | 287A | LEU | 2.54 | 3.36 | 140.88 | ✓ | × | 2197 [Nam] | 2625 [O2] |
| 33 | 288A | GLU | 3.53 | 3.97 | 109.97 | × | ✓ | 2866 [O3] | 2211 [O3] |

π-Stacking

| Index | Residue | AA | Distance | Angle | Offset | Stacking Type | Ligand Atoms |
|---|---|---|---|---|---|---|---|
| 1 | 126A | TYR | 3.83 | 3.99 | 1.60 | P | 2790, 2791, 2792, 2793, 2795, 2796 |

π-Cation Interactions

| Index | Residue | AA | Distance | Offset | Protein charged? | Ligand Group | Ligand Atoms |
|---|---|---|---|---|---|---|---|
| 1 | 5A | LYS | 3.80 | 1.86 | ✓ | Aromatic | 2856, 2857, 2858, 2859, 2861, 2862 |

Salt Bridges

| Index | Residue | AA | Distance | Protein positive? | Ligand Group | Ligand Atoms |
|---|---|---|---|---|---|---|
| 1 | 4A | ARG | 5.46 | ✓ | Phosphate | 2848, 2848, 2849, 2850, 2851, 2847 |
| 2 | 217A | ARG | 3.33 | ✓ | Phosphate | 3208, 3208, 3207, 3209, 3210, 3211 |
| 3 | 236A | LYS | 2.59 | ✓ | Phosphate | 3055, 3055, 3057, 3058, 3056 |
| 4 | 236A | LYS | 5.06 | ✓ | Phosphate | 3035, 3035, 3034, 3036, 3037, 3038 |

**Figure.S2** PLIP interactions output of MApta$^{pro}$-IR1-M$^{pro}$ complex

Hydrogen Bonds

| Index | Residue | AA | Distance H-A | Distance D-A | Donor Angle | Protein donor? | Side chain | Donor Atom | Acceptor Atom |
|---|---|---|---|---|---|---|---|---|---|
| 1 | 123A | SER | 1.89 | 2.77 | 146.34 | × | × | 2570 [Npl] | 946 [O2] |
| 2 | 123A | SER | 2.08 | 2.81 | 131.44 | ✓ | ✓ | 944 [O3] | 2591 [Npl] |
| 3 | 123A | SER | 2.38 | 2.81 | 106.28 | × | ✓ | 2591 [Npl] | 944 [O3] |
| 4 | 139A | SER | 2.14 | 2.89 | 133.35 | ✓ | ✓ | 1066 [O3] | 2628 [O3] |
| 5 | 139A | SER | 2.13 | 2.94 | 140.23 | × | ✓ | 2620 [O3] | 1066 [O3] |
| 6 | 139A | SER | 3.08 | 3.70 | 121.72 | × | × | 2616 [Nar] | 1068 [O2] |
| 7 | 139A | SER | 1.81 | 2.78 | 165.18 | × | × | 2615 [Npl] | 1068 [O2] |
| 8 | 274A | ASN | 3.10 | 3.67 | 118.57 | ✓ | ✓ | 2113 [Nam] | 2856 [O3] |
| 9 | 274A | ASN | 3.49 | 3.92 | 108.91 | × | ✓ | 2833 [O3] | 2112 [O2] |
| 10 | 276A | MET | 2.07 | 3.03 | 163.06 | × | × | 2782 [Npl] | 2127 [O2] |
| 11 | 277A | ASN | 2.01 | 2.93 | 153.96 | ✓ | ✓ | 2133 [Nam] | 2800 [Nar] |
| 12 | 277A | ASN | 2.04 | 2.93 | 149.81 | × | ✓ | 2803 [Npl] | 2132 [O2] |

π-Cation Interactions

| Index | Residue | AA | Distance | Offset | Protein charged? | Ligand Group | Ligand Atoms |
|---|---|---|---|---|---|---|---|
| 1 | 279A | ARG | 4.63 | 0.82 | ✓ | Aromatic | 3039, 3040, 3041, 3042, 3044, 3045 |

Salt Bridges

| Index | Residue | AA | Distance | Protein positive? | Ligand Group | Ligand Atoms |
|---|---|---|---|---|---|---|
| 1 | 4A | ARG | 4.89 | ✓ | Phosphate | 2690, 2690, 2689, 2691, 2692, 2693 |
| 2 | 5A | LYS | 3.93 | ✓ | Phosphate | 2667, 2667, 2666, 2668, 2669, 2670 |
| 3 | 222A | ARG | 3.90 | ✓ | Phosphate | 3095, 3095, 3097, 3098, 3094, 3096 |

**Figure.S3** PLIP interactions output of MApta$^{pro}$-IR2-M$^{pro}$ complex

Hydrogen Bonds

| Index | Residue | AA | Distance H-A | Distance D-A | Donor Angle | Protein donor? | Side chain | Donor Atom | Acceptor Atom |
|---|---|---|---|---|---|---|---|---|---|
| 1 | 131A | ARG | 3.10 | 3.52 | 107.32 | ✓ | ✓ | 975 [Ng+] | 2576 [O3] |
| 2 | 137A | LYS | 2.03 | 2.98 | 165.01 | × | ✓ | 2576 [O3] | 1026 [N3] |
| 3 | 137A | LYS | 2.10 | 3.06 | 164.45 | × | ✓ | 2572 [Nar] | 1026 [N3] |
| 4 | 137A | LYS | 3.09 | 3.79 | 126.55 | ✓ | ✓ | 1026 [N3] | 2571 [Npl] |
| 5 | 137A | LYS | 3.02 | 3.79 | 135.34 | × | ✓ | 2571 [Npl] | 1026 [N3] |
| 6 | 138A | GLY | 2.96 | 3.73 | 135.36 | ✓ | × | 1029 [Nam] | 2600 [O3] |
| 7 | 138A | GLY | 2.44 | 3.26 | 141.62 | × | × | 2603 [O3] | 1032 [O2] |
| 8 | 139A | SER | 1.91 | 2.76 | 142.05 | ✓ | × | 1033 [Nam] | 2928 [O2] |
| 9 | 139A | SER | 3.00 | 3.95 | 165.55 | ✓ | ✓ | 1036 [O3] | 2906 [O3] |
| 10 | 139A | SER | 3.46 | 3.95 | 113.65 | × | ✓ | 2906 [O3] | 1036 [O3] |
| 11 | 191A | ALA | 2.76 | 3.23 | 110.94 | × | × | 3159 [O3] | 1427 [O2] |
| 12 | 196A | THR | 3.09 | 3.47 | 104.58 | ✓ | ✓ | 1458 [O3] | 2526 [Nar] |
| 13 | 238A | ASN | 2.01 | 2.98 | 165.99 | ✓ | ✓ | 1782 [Nam] | 2502 [Nar] |
| 14 | 239A | TYR | 2.32 | 2.85 | 113.99 | × | ✓ | 2530 [O3] | 1792 [O3] |
| 15 | 274A | ASN | 3.69 | 4.00 | 101.39 | ✓ | × | 2058 [Nam] | 2291 [O3] |
| 16 | 275A | GLY | 3.12 | 3.59 | 111.12 | ✓ | × | 2066 [Nam] | 2291 [O3] |
| 17 | 287A | LEU | 1.82 | 2.78 | 162.66 | ✓ | × | 2127 [Nam] | 2533 [O2] |
| 18 | 288A | GLU | 3.09 | 3.88 | 140.12 | × | ✓ | 2556 [O3] | 2140 [O2] |

Salt Bridges

| Index | Residue | AA | Distance | Protein positive? | Ligand Group | Ligand Atoms |
|---|---|---|---|---|---|---|
| 1 | 4A | ARG | 5.46 | ✓ | Phosphate | 2967, 2967, 2969, 2970, 2966, 2968 |
| 2 | 5A | LYS | 4.64 | ✓ | Phosphate | 2578, 2578, 2577, 2579, 2580, 2581 |
| 3 | 5A | LYS | 3.68 | ✓ | Phosphate | 2947, 2947, 2946, 2948, 2949, 2950 |

**Figure.S4** Principal Component Analysis plot for the 100ns trajectories with instantaneous conformations (trajectory frames) colored from black to green in order of time. MApta$^{pro}$-M$^{pro}$ (A), MApta$^{pro}$-IR1-M$^{pro}$ (B), and MApta$^{pro}$-IR2-M$^{pro}$ (C).

A

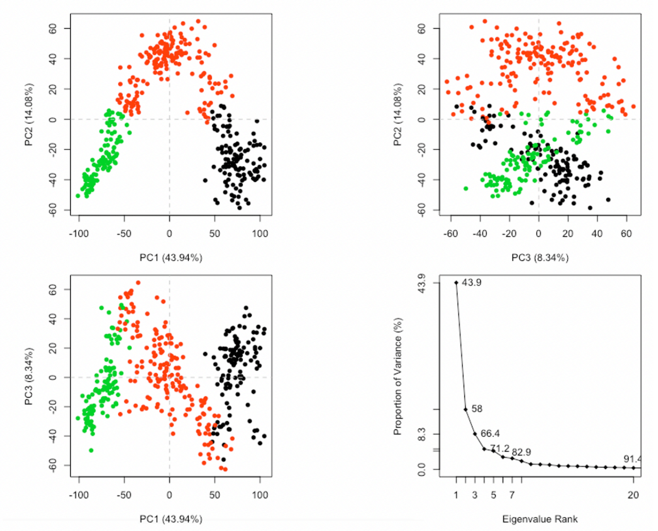


B

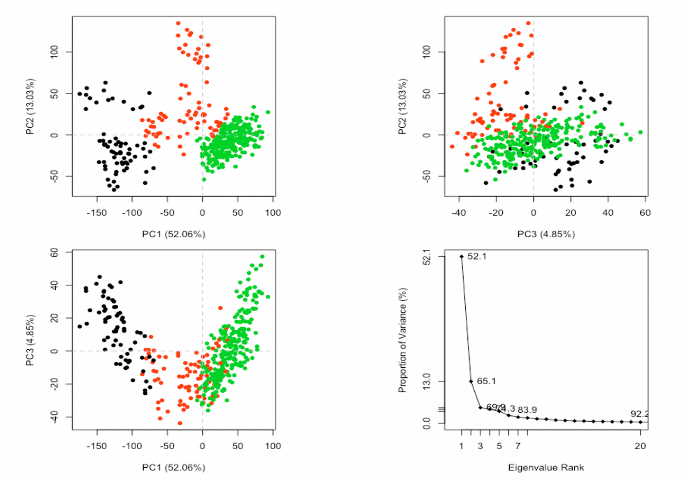


C

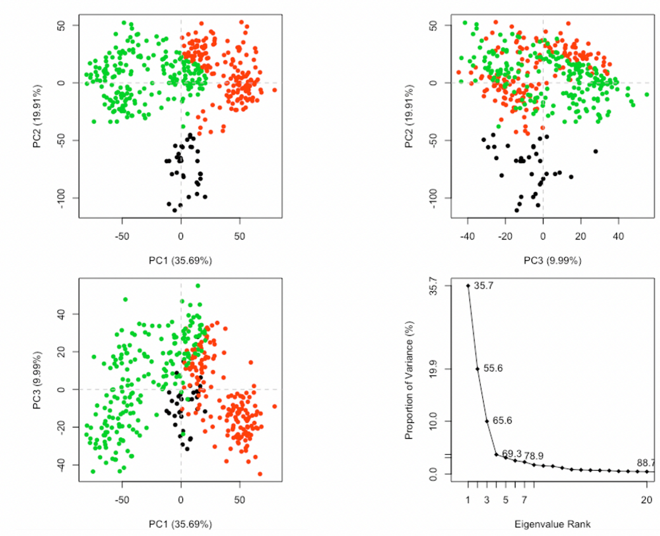

**Output of RNAinverse WebServer**

Results have been computed using RNAinverse 2.4.13. An equivalent command line call would have been
RNAinverse -Fmp -f 0.5 -R 10 -d2 < seqstruct.txt > inverse.out

RNA parameters are described in
Mathews DH, Disney MD, Childs JL, Schroeder SJ, Zuker M, Turner DH. (2004) Incorporating chemical modification constraints into a dynamic programming algorithm for prediction of RNA secondary structure. Proc Natl Acad Sci U S A 101(19):7287-92.

If you find these results helpful for your work you may want to cite:

Gruber AR, Lorenz R, Bernhart SH, Neuböck R, Hofacker IL.
The Vienna RNA Websuite. Nucleic Acids Res. 2008
Lorenz, R. and Bernhart, S.H. and Höner zu Siederdissen, C. and Tafer, H. and Flamm, C. and Stadler, P.F. and Hofacker, I.L. "ViennaRNA Package 2.0", Algorithms for Molecular Biology, 6:1 page(s): 26, 2011

The RNAinverse Web Server found the following sequences that have a MFE structure identical to your target structure:

| Sequence | Energy | Link to RNAfold Server |
|---|---|---|
| GGCCGAGGAGCGGUGUGGCAGGGCUGAAGACCAUAUGGGC | | submit |
| GGCAGCGGAGCGAUGUGGGAGGCCUGAAGAUCAUAGGUGC | | submit |
| GGCGGCGGAGAGGUGGGACAGGGUCGACGGCCGGAGGUGC | | submit |
| GGCGGCGGACGUGUGGGCUCGGAGCGACGGCAAAAGGUGC | | submit |
| GGUGGGGGAGAGACGGGCCCGGGGUGACGGUCAUAUGCAC | | submit |
| GGCGAGGGAGGGUCGUGGGCAGCCUGAAGGACAUACGUGC | | submit |
| GGCGGCGGAGCGGUGCAGCAGGGCUCAGGACCAUAGGUGC | | submit |

Note: You can click on table header to sort results.

GGCGGCGGAGGUUCGGGCCCGGGGUGACAGAGAUAGGUGC 14 d= 1
CGCUGCGAAAGGCCACUCCAAAGGAAUGCGGCAAAGGAGC 26 (0.480504)
GGCGGUUGAGCGGUGUGGGCGUCCUGAAGACCAGAAGUGC 10 d= 1
CGCAGGUAAAAGGAGGGCCUAAGGUGACGUCCGAUCGUGC 23 (0.457527)
GGCUCCGGAGCGUGGUGGGCGGCCUGAAUUACAUAGGGGC 8 d= 2
CGGGUCGAAAAGGUACAGCAAAGCUAAGAACCGCAGUCCC 25 (0.374643)
GGCGGCGGAGCGAUGUUGACGGUCACAGGGUCAUAGGUGC 11 d= 5
CGGUGUGAAAAGUCGCUGCAAGGCACUGGGACGGAAGACC 28 (0.468169)
GGAGGCGGAAUUCUGAUGCCGGGCAGAUGAGAAAAGGUUC 19 d= 2
GGGUGCGGAGGACUGAGGCAAUGCCAAUGAGUGAAGGGCC 24 (0.478094)
GGCGGCGGAGCGGGGUCAUAGAAUGAAAGCCCAUAGGUGC 9 d= 2
CCCGGCGAAAACAGGCCCCAAUGGGAAGGCUGGAAGGCGG 26 (0.514584)
GGCUGCGUUGCGGUGUUUGGGGCAAGAGGACCUUGGGAGC 16 d= 5
UGCUGCAAUAUGCUGGAGGGAACCUAACGAGCUCGGGAGC 25 (0.446568)
GGCCGAGGAGCGGUGUGGCAGGGCUGAAGACCAUAUGGGC 11
UGCUGCGAAACGCUGGGGCGAAGCCGACGAGCAAAGGAGC 20 (0.469916)
GGCACGGGAUCGGUGAGCUCGGAGCGAUGGCCAAAUGUGC 16 d= 2
CGCCGCGGAAGCUUGGACCAAUGGUAACGGAGAAAGGGGC 24 (0.520218)
GCCGGGGGAGAACGGAGUCUGGGACACUGCGUAUAUGUGG 20 d= 5
ACCGGGGAGUAGCCGAACCUAUGGUACUGGGCGAACGCGG 29 (0.517762)
GGCGGGGGAUCGGUGGGCGCGGCGCGAUGGCCAAACGUGC 13 d= 6
GCCAGGGAAAGGAUGCGCGGAUCGCAAGGGUCAAACGUGG 24 (0.54068)
GGCGGCGGAGCGAGGGUCCCGGGGAGGUUUUCAUAGGUGC 12 d= 6
AGCAGGGAAACGAGGAGACAAAGUCAAUGCUCAAACGUGC 20 (0.481398)
GGCGGCGGAUAAGCGUCUCGGGGAGUAAGGCUAUAGGUGC 15 d= 1
GGCUGCGAAAAUGCGAACCAAAGGUCAUGGCAAAAGGAGC 22 (0.47483)
GGCAGCGGAGCGAUGUGGGAGGCCUGAAGAUCAUAGGUGC 9
CGCAGCGAAAGGACGUGGGCUCCCUGAAGGUCAAAGGUGC 15 (0.293968)
GGCGGCGUAUAUCCGGGGCCGGGCUGAUUGGACUAGGUGC 15 d= 1

CGCUGCGAAAACCCACGCCAAUGGCAAGCGGGGAAGGAGC 27 (0.470274)
GGCGGCGGUCGGUAUUGGCAGGGCUUAAGUGCAUAGGUGC 14 d= 1
AGGUGGGAACGGCAUUUCCAAAGGACCAUUGCAAACGACC 27 (0.127829)
GGCGGUUUAACAGCGGGCCCGGGGCGACGGCUACAAGUGC 18 d= 2
AGCAGGGAAAGAGCGCGCCCAGGGCAAGGGCUACACGUGC 22 (0.74035)
GGCGGAAGAUAGGUGGACUCGGAGUGACGGCCGUAUGUGC 16 d= 4
AGCUGGGGAAAAGGGGACGCAACGUAACGCCUGAACGAGC 21 (0.468629)
GGCGGCGGAGAGGUGGGACAGGGUCGACGGCCGGAGGUGC 13
CGCAGCGAAAAGAUGGGACAAAGUCAACGGUCGGAGGUGC 22 (0.471478)
GGCGGCAAAUCGGCGGGCUAGGAGCGACGGCCACAGGUGC 15 d= 1
AGCUGCGAACAGGUGCGCUUAAAGCAAGGGCCGAGGGAGC 23 (0.520601)
GGCGGUGGAAAGGGGAAUGCGGCAUGAUGCCCGUAGGUGC 11 d= 1
AGCAGUGAAAAGAGGCACGGGACGUAAGGCUCGAAGGUGC 20 (0.469399)
GGAGGCGUAGGGUAGUUGGCGGCCAGAAGUGCAUAGUUUC 14 d= 1
CGGCCCGAAAAGGAGGGGCAAAGCCAGCGUCCGAAGAGCC 26 (0.525626)
GGCGAGGGAUUUCGGUUGGCGGCCAGAAGCGAGUACAUGC 16 d= 1
AGCGAGGAAAAUCGGCUGGAACCCAUUGGCGAGAACGUGC 25 (0.493144)
GGCGGCGGAGGAUUCGGGCCGGGCUCAUGAGUAUAGGUGC 15 d= 3
AGGAGCGAAAAGCCACAGCAAAGCUAAGCGGCGAAGGUCC 25 (0.560869)
GGCAGUGGAGCGGCGGUCCCGGGGAAAUGGCCAUAGGUGC 14 d= 1
GGCGGCGAAACGGAGCUCCCAGGGAAUGGUCCAAAGGCGC 18 (0.5363)
GGCGGCGGAGAGUUGGUGACGAUCAGAUGGACAUAGGUGC 13 d= 4
AGCGGGGGAGAGCAGCGGACAAUCCAAGGUGCGGACGCGC 20 (0.531532)
GGCAGUUGAGCGGGGUGCCCCAGGUGAGGCCCAUAGGUGC 11 d= 2
ACCGGAUACAAGAAGCGCCUAAGGCCAGGUUCGAUUGCGG 30 (0.500744)
GACGGCGGAAUGAGGGGCCCGGGGUUAUGUUCAGAGGUGU 16 d= 1
GGCUGCGGAAAGAUGGGCCCAAGGUUACGGUCGGAGGAGC 20 (0.472142)
GGCGGGGGAACGAUGGGCCCGGGGUGACGGUCGAACGUGC 16 d= 1
AGCAGGGAAAGGAUGGGCCAAGGGUAACGGUCAAACGUGC 22 (0.568182)
GGCGGCGGAACGGUGUUGAGGGUCAUAAAGCCAUAGGUGC 10 d= 1
GGCCGCGCACAGCCGCUGGACACCAUUGGGGCAUAGGGGC 21 (0.454439)
GGCGGCGGGAGUCUGGGUCCGGGACGAUGGGAGGAGGUGC 19 d= 1
CGCGGCGAAAAUGUGAGCCUAGGGCAAUGACAGAAGGUGC 21 (0.479911)
GGCUCUGGAGCGGUGUGGCCUAGCUGAAUGCCAUAAGGGC 12 d= 2
ACGCCCGACAUGGCCCUGCACAGCAUUGAGCCUAAGAGCG 31 (0.209772)
GGUGGCGGAGAGCUGAGCCCGGGGUGAUGAGCAUAGGUAC 14 d= 1
GGUCGCGGAAAGCUGGGCCCAGGGUGACGAGCGGAGGGAC 20 (0.4703)
GGCGGCGGAAAGGUGGGCUCGGAGCGACAGCCAAAGGUGC 13 d= 1
CGCUGCGAAAAGCUGGACCAAAGGUGACGAGCAAAGGAGC 22 (0.537508)
GGCGGCGGAGGACAGGGGCGGGGCUGAUUUGUAUAGGUGC 13 d= 1
CGGCGCGAAAAUGCGAGGCACAGCCAAUGGCAGAAGGGCC 25 (0.548532)
GGCGGCGAAGCGGGUAGGCCGGGCCGAUUCCCAUAGGUGC 8 d= 1
ACCUGGGAAAAGUCACGCCAGCGGCACGCGACAAACGAGG 28 (0.525087)
GGGUGCGGAGAGGUGAGCUCGGAGCGAUGGCCAUAGGACC 15 d= 1
AGCAGGGAAAAGGUGAGCGCAACGCAAUGGCCGCACGUGC 21 (0.473032)
GGCGGCGGAGCGGCGUUCCCGAGGAUAAGGCCAUAGGUGC 11 d= 1
AGCAGGGAACCGGCGCUCCAAAGGAUUGGGCCAUACGUGC 22 (0.578169)
GGCGGCGGAGCGGGGGGCCCCAGGUGAUUCCCAUAGGUGC 8 d= 1
AGCAGGGAAAAGGGCGUCCAAAGGAAUCACCCGAACGUGC 24 (0.472368)
GGCGGCGGAGCGGUUGUCCCGGGGAAUCGACCAUAGGUGC 14 d= 1
ACCUGCGAAAGCCGCGUCCAAAGGAAUCACGGAAAGGAGG 28 (0.458049)
GGCGGUGGACAGGUAGUCAGGGUGAGACGGCCAUAGGUGC 15 d= 5
AGCAGUGAAAAGAUGGGGCAAAGCCGACGGUCGAAAGUGC 23 (0.495481)
GGCGGCGGAGUGGAGGGGCCGGGCUCAUGUCUAAAGGUGC 12 d= 3
GCCGGGGAACAAGGGGGGCCGAGCCAACGCCUAAGCGCGG 21 (0.455853)
GGCGAGGGAGAAGCGAGUCCGGGACGAUGGCUAUACGUGC 16 d= 1
ACCAGGGAAAAUGUGAGCCCCCGGCAUUGGCAGAACGUGG 27 (0.461701)
GGCUGCGGAGAGCAGGUUGCGGCAAGACGUGCAGAGGAGC 16 d= 5

AGCUGGGAAAAGGAGGCGGUGACCGAACGUCCAAACGAGC 21 (0.590066)
GGCAGCGUUGCGGUCUGGGCGGCCUGAAGGCCUUAGGUGC 10 d= 1
CGCCGCGAACUGGAACAGCAAAGCUAAGAUCCUAAGGGGC 22 (0.403182)
GCACGCGGAGCGGCGAGGUCGUACCUAUCGCCAUAGGGUG 17 d= 1
GACCGCGAAAAGUCACAGCCAAGCUAAGCGACAGAGGGGU 23 (0.468679)
GGCGGCGGAGCGGAGGGCUCGUAGUUUCUUCCAUAGGUGC 11 d= 1
AGCGGGGAAAAGGGCGUCCAAAGGAAUCACCCGAACGCGC 24 (0.463013)
GGCAGCGGAGCGUCGGCCACGGUGGGACGGACAUAGGUGC 13 d= 5
CGCCGCGCAGACUGGGGCCCAAGGCGACGCAGAAAGGGGC 20 (0.48679)
CGCGGCGGAGCAGAGGGACCCGGUUGACGUCUAUAGGUGC 12 d= 3
AGCUGCGAAACAGAGGGCCCUGGGUAACGUCUAAAGGAGC 19 (0.474945)
GGCUAGGGAGUUCUGGGCCGGGGGCGACGAGAGAACGAGC 23 d= 1
GGCCACGAACUGCAGGGGCCCAGCCAACGUGCUAAGCGGC 23 (0.455683)
GGCGGCGGAGCGGGGGGGCGGCCUGAUACCCAUAGUUGC 6 d= 2
CGGCGCGAACGAGGCGAGCAAUGCUAACACCUAAAGGGCC 23 (0.423472)
GGCGGCGGACGUGUGGGCUCGGAGCGACGGCAAAAGGUGC 15
GGCGGCGAAAGUGAGGGCCCAAGGCGACGUCAGAAGGUGC 19 (0.458444)
GGUGGCGGAGCGGGUGCCUCGGAGGAACGCCCAUAGGUAC 13 d= 1
CGCCGCGAAAGCUGGAACCCAAGGUAAUGCAGAAAGGGGC 22 (0.527092)
GGCGGAGGAGAGGGGGGGCCGGGCUGACACCCGUAUGUGC 10 d= 1
AGGUGCGGAUAGGGCGACCAUAGGUAACACCCGAAGGACC 22 (0.453653)
GGCGGCGGAGCGCUCGUGACGGUCAGAUGGGCAUAGGUGC 12 d= 5
ACCAGCGAAAAUCCACAGCCAAGCUAAGCGGAGAAGGUGG 26 (0.588895)
GGCGGCGGAGCGGAGGGAUGAGAUCGAUGUCCAUAGGUGC 11 d= 1
CGCGGCGAAGAGGAGAAGCAAUGCUCAUGUCCGAAGGCGC 19 (0.445509)
GGCGGCGGAGCGGCGUCUCCAGGAGUAAUGCCAUAGGUGC 10 d= 1
CGCAGCGAAAACGGGCUCCCAUGGACUGGCCGGAAGGUGC 22 (0.618813)
GGCGGAGGAAACCGAAUGGCGGCCAGAUACGGAUAUGUGC 16 d= 1
AGCAGCGGAAACCCACAGGCGGCCUAAGCGGGAAAGGUGC 19 (0.472506)
GCCGGCGGAACAUUGGAGGCGGCCUCGUGAGUGUAGGUGG 18 d= 4
UCCAGGGAAAAGUGGCGGUUAAACCAAGGCACGAACGUGG 24 (0.591054)
GGCGGGGGAGAGGAGUUGGCGGCCAGAAGUCCAUAUUUGC 11 d= 1
ACCGGGGAAAAGGUGGUGGUAUCCAGUCGGCCGAACGCGG 24 (0.433321)
GGCGGCGGAACUAGGGGCUCGGAGCGACGUUAGAAGGUGC 16 d= 1
AGCUGGGGAAUUGAGGGCCAGAGGCGACGUCAUAGCGAGC 24 (0.467282)
GGCGGCGGAUUGGAGGGGCCGGGUCGAUGUCCACAGGUGC 11 d= 2
CGCGGCGAAUUGGAGCGACUCGGUCAAGGUCCUAAGGUGC 18 (0.495283)
GGCGGCUGAUUGGUGGCGCGGGGUGAAUGGCCAGUGGUGC 16 d= 4
CGCCGCUGAAGUGCGAACCAAAGGUAAUGGCAAAUGGGGC 24 (0.436545)
GGAUGCGGAGCGGGGAUGGCGGCCAGAUGCCCAUAGGAUC 11 d= 1
CGCAGGGAAACGGAGAACGAAGCGUAAUGUCCAAACGUGC 19 (0.475657)
GGCGGUGGAGGUUCCAGGACGGUCUGAUGGGAAGAGGUGC 15 d= 1
CGCAGGGAAAAGGGCGAGCAGAGCUAACACCCGAACGUGC 20 (0.46217)
GGCGGCGGAAAGAACCGGGCGGCCUGUGGUUCGAAGGUGU 16 d= 1
AGCGGGGAAAAGGGGGAGCAAAGCUAACGUCCGAACGUGC 20 (0.4849)
GGCGGCGGAUUAGGGAGCCCGGGGUUAUGUCUAUAGGUGC 13 d= 1
CGCUGCGAAAACCGGAACCAAAGGUAGUGCGGGAAGGGGC 25 (0.520842)
GGCGGCGGAAGGCAGAGGUCGGGCUGAUCUGCAUAGGUGC 12 d= 1
CGCCGCGAAAAGUCACAGCAUAGCUAAGCGACCAAGGGGC 24 (0.525635)
GACGGGGAAUUUUGGCGGCCGGGCUUAGGCGAAUGUGCGU 20 d= 1
GGCGGGGAACUUCGGCGCCCAAGGCAAGGCGAUAGCGCGC 24 (0.461979)
GGCGGCGGAAAGGUGGAUGGGGCGUGAUGACUGAAGGUGC 15 d= 1
GCCUGGGAAAAUGUGCCCGAAGCGGAAGGACAGAACGAGG 26 (0.634141)
GCCGGCGGACCCUGGUAGGCAGCCUCAAGCAGAUAGUUGG 14 d= 1
CGCAACGAAAUCCGGCACCAAUGGUCAGGCGGCAAGAUGC 25 (0.483062)
GGCGGGGGAGACGUGUAGGCGGCCUUAAGACGAUAUGCGC 13 d= 1
AGCCGAGAAAACGUGCAGCCAAGCUCGGGACGGAAUGGGC 25 (0.497232)
GACGGUUGAGCGGUGUGGGGGUCCUGAAGACUAUAAGUGU 13 d= 2

CGGCGAUGAAAGGAGCGCCCAGGGCAAGGUCCGAUUGGCC 25 (0.527823)
GGCGGCGGAGCGGUGUUCCCGGGGAGAGGGCCAUAGGUGC 10 d= 5
ACCGGGGAAAAGGAGGGCCAAAGGCAUCGUCCGAACGCGG 26 (0.474848)
GGCGGCGGAGAGGAUAAAGCGGCUUGAUGUCCAAAGGUGC 11 d= 1
CGCCGCGAAAAGGAACAGCCAAGCUAAGAUCCGAAGGGGC 21 (0.416564)
GGCGGCGGAGCGGUGUUCCCGGGGAGAGGGCCAUAGGUGC 10 d= 5
UGCGGGGAAGAGGUGGACCCAAGGUAACGGCCGAACGUGC 20 (0.544199)
GGUGGCGAAACGCGGAGGCCGGGCCGAUGUGCAUAGGUGC 13 d= 8
GGGCGCGAAACGCUGAGGCAAAGCCAAUGAGCAUAGGGCC 21 (0.451894)
GGCUUCGGAUUUGGGGUGCCGUGCAGUCGCCAAUAGUAGC 18 d= 1
CGCGUCGAAGUCGGGGGGCAAAGCCAGCGCCGUAAGUCGC 22 (0.452236)
GGUGGUGGAACUUUGAGGCCGGGCUGAUGGGGAUAGGUAC 16 d= 7
AGCAGGGGAAACCGGGACCUAGGGUAACGCGGGAACGUGC 23 (0.535458)
GGCGGCGGAGUCGUAAAGUCGGGCUGAUUACGUGAGGUGC 14 d= 1
CGGCGCGAACUGCGAGACCAAAGGUAACAUGCUAAGGGCC 26 (0.301078)
GGCGGCGGAGCGGUGGUGUCGAGCAAACGGCCAUAGGUGC 12 d= 4
AGCAGGGAACAGGUGGAGCUAAGCUAACGGCCAGACGUGC 21 (0.465807)
GCCAGUAGAGCGGGUGUGUCGGACGGUUUCCCAAAGGUGG 15 d= 1
ACCAGGAAAAACCGUCCGCAAAGCGUCGUCGGAAGCGUGG 29 (0.447044)
GGCGGCCAAGCGUAGUGCCCGGGGUGAAGUACAAAGUUGC 13 d= 1
ACCACCGAAAAGACGCGCCCAUGGCAAGGGUCGAAGAUGG 26 (0.474508)
GGUGGGGGAGAGACGGGCCCGGGGUGACGGUCAUAUGCAC 17
ACCGGGGAAAAGCCGUGCCACAGGUGAAGGGCGGACGCGG 23 (0.457899)
GGCGGCGGAGCGGAAAGGGCGUCCUGAUUUCCAUAGGUGC 8 d= 1
CGCCGCGAAAAGGAACAGCCAAGCUAAGAUCCGAAGGGGC 21 (0.416564)
GGCGGCGGAGUGGUGUGACGGGGUUGAAGACCUAAGGUGC 10 d= 3
ACCUGCGAAUUGGAGUGGCGUAGCUGAGGUCCUAAGGAGG 20 (0.399865)
GAGCGUGGAUGGGCGUGCCCGGGGUGAGAGUCGCAGCGUU 21 d= 2
AGGCUCGAAAAGGUGCACCAAGGGUUAGGAUCGUAGUGCC 25 (0.464536)
GGCAGUGGACGUGUGGGCUCGGAGCGACGGCAUUAGGUGC 17 d= 1
GGCUGCGAAAUUGAGGGCCCAGGGCGACGUCAUAAGGAGC 20 (0.470232)
GGCGGCGGAGGAGCGGGCCCGGGGUGAUUGUUAAAGGCGC 14 d= 1
AGCGGCGGAAAUCCACACCUGGGGUAAGCGGAGAAGGCGC 23 (0.581186)
GGACGCGAAGAGCUAGAGGUGGCCUGAUCGGCAUAGGGUC 18 d= 1
CGGUGGGAAAAGGAACAGCAAAGCUAAGAUCCGAACGGCC 26 (0.421006)
GGCGGCGGAACGGAGUGAACUAUUUGAAGUCCAUAGGUGC 8 d= 3
CGCCGCGAAACGGAGCGCUCAAAGCUAGGUCCACAGGGGC 19 (0.529792)
GGUGGCGGACGAGUGCUUUCGGGAAAGGGGCUAUAGGUAC 19 d= 6
CGCCGUGAAAGCCAGUACCAAUGGUCAAGUGGAAAAGGGC 25 (0.462718)
GGCGAGGGAGGGUCGUGGGCAGCCUGAAGGACAUACGUGC 12
AGCAGGGAAAGGUCGCUGGCAACCAUUGGGACAAACGUGC 23 (0.611359)
GGCGGGGGAUAGGAUUGCCUGGGGUGAGGUCCGUAUGUGC 15 d= 1
AGCAGGGAAAAGGAGUGGCGAAGCUGAGGUCCGAACGUGC 19 (0.448948)
GGCGGCGGAGCGGUGCAGCAGGGCUCAGGACCAUAGGUGC 11
GGCGGCGAAAGGGUGCAGCAAGGCUCAGGACCAUAGGUGC 15 (0.497669)
GGCGGCGGAGUAGUGGUCCCCGGGAGUCGGCUAAAGGUGC 17 d= 1
CGCAGGGACAUGGCGCUCCACAGGAUUGGGCCUAACGUGC 26 (0.668883)
GUCGGGGGAGGGCUGAUGCCGGGCAGAUGAGCAAACGUGA 18 d= 1
ACCGGGGAAAAGGUGCUGGAAUCCAUUGGGCCGCACGCGG 25 (0.621481)
GCCGGGGGAGGGGCUUGGCCGGGCCGAAAGUCGAACGUGG 16 d= 1
ACCGGGGAAAGGACGCAGCCAGGCUAAGGGUCAAACGCGG 23 (0.721015)
GCCGGGGGAGUGAUGGGGGCGGCCUGACGAUCGAACGUGG 16 d= 1
UCCGGGGCACUGAUGGAGGCAACCUAACGAUCUCACGCGG 24 (0.471734)
GGCGGCGGUGCGGACGGAGCGGCUCUACGUCCAUAGGUGC 11 d= 1
AGCUGCGAAAGCCGCGUCCAAAGGAAUCACGGAAAGGAGC 26 (0.454422)
